# Local Pharmacological Interventions for Pain Relief During Peripheral Venous Cannulation: A Systematic Review with Implications for Clinical Nursing Practice

**DOI:** 10.3390/jcm15031262

**Published:** 2026-02-05

**Authors:** Aleksandra Maruszak, Damian Romańczuk, Sandra Lange, Wioletta Mędrzycka-Dąbrowska, Grzegorz Cichowlas, Anna Gąsior

**Affiliations:** 1 Department of Anesthesiology and Intensive Care, University Clinical Center, Dębinki 7, 80-952 Gdańsk, Poland; a.maruszak@gumed.edu.pl (A.M.); damian.romanczuk@gumed.edu.pl (D.R.); 2 Department of Anesthesiology and Intensive Care, Medical University of Gdańsk, Dębinki 7, 80-952 Gdańsk, Poland; 3 Department of Internal and Pediatric Nursing, Medical University of Gdańsk, Dębinki 7, 80-211 Gdańsk, Poland; langa94@gumed.edu.pl; 4 Department of Anesthesiology and Intensive Care Education, Medical University of Warsaw, 02-097 Warsaw, Poland; grzegorz.cichowlas@wum.edu.pl; 5 Department of Anesthesiology and Intensive Care, Czerniakowski Hospital sp. z.o.o., 00-739 Warsaw, Poland; anna.gasior@szpitalczerniakowski.waw.pl

**Keywords:** pain, vascular cannulation, local analgesia, pharmacological analgesia

## Abstract

**Background/Objectives:** Peripheral venous cannulation is one of the most frequently performed invasive procedures in adult patients and is often associated with procedural pain. Despite the availability of various pain-reduction strategies, analgesia during cannulation is not consistently implemented in routine clinical practice. The aim of this review was to identify local pharmacological interventions recommended for reducing procedural pain during peripheral venous cannulation, to assess their clinical effectiveness, and to determine which interventions may be feasibly incorporated into everyday clinical practice. **Methods:** A literature review was conducted including randomized controlled trials, prospective studies, and meta-analyses involving adult patients undergoing peripheral venous cannulation. Outcomes of interest included procedural pain intensity, clinical effectiveness of pharmacological interventions, and their impact on additional outcomes such as patient satisfaction, anxiety, and safety. **Results:** Peripheral venous cannulation was most commonly associated with moderate-intensity pain. The use of local pharmacological interventions—particularly vapocoolant sprays and topical local anesthetics in cream or patch form—resulted in a significant reduction in pain intensity compared with placebo or no intervention. Several studies also reported improved patient satisfaction and a favorable safety profile of the analyzed interventions. **Conclusions:** Current evidence indicates that local pharmacological interventions are effective and safe in reducing pain associated with peripheral venous cannulation and may represent a valuable component of patient-centered clinical care. In addition to summarizing clinical effectiveness, this review highlights practical and organizational factors influencing the implementation of pharmacological pain-reducing interventions in routine nursing practice.

## 1. Introduction

Intravenous catheter insertion is one of the most frequently performed procedures in hospitals and outpatient clinics, becoming a standard component of patient care. Although the procedure is considered routine by medical staff, for many patients it represents a significant source of stress, anxiety, and pain. Unpleasant experiences associated with cannulation may affect not only the patient’s subjective comfort but also their cooperation during the procedure, consequently influencing its effectiveness and safety. Procedural pain may trigger physiological responses such as increased heart rate, elevated blood pressure, and increased cortisol levels. For this reason, effective pain management and stress-reduction strategies are essential in clinical practice. Both pharmacological and non-pharmacological methods are used to divert attention and minimize the procedural pain associated with cannulation. The choice of an appropriate method depends on the type of procedure, the patient’s age, clinical condition, and the availability of resources [1,2,3,4,5,6]. The use of pharmacological local-anesthetic methods enables effective reduction in procedure-related pain; however, their selection depends on factors such as onset and duration of action, method of administration, potential adverse effects, availability of agents, and individual patient preferences. Additionally, effective pain control contributes to improved hospitalization experiences, enhances patient trust in medical staff, and may reduce the development of anxiety related to future medical procedures. In recent years, increasing attention has been directed toward studies comparing different local anesthesia strategies and non-pharmacological techniques in order to identify the most effective and best-tolerated approaches for patients [7,8,9,10,11,12]. Although several systematic reviews and meta-analyses have evaluated the efficacy of vapocoolants and topical anesthetics, limited attention has been given to how these interventions can be implemented in routine clinical and nursing practice across different healthcare systems. In particular, evidence addressing organizational constraints, staff training, and availability of pharmacological agents in Central and Eastern European settings remains scarce [13,14,15,16]. This review aims not only to synthesize recent clinical evidence (2015–2025) but also to highlight practical and contextual factors influencing the feasibility of pain-reducing interventions during peripheral venous cannulation.

### Aim

The aim of this review is to identify which local pharmacological interventions are recommended during peripheral venous cannulation to reduce procedural pain, to evaluate their clinical effectiveness, and to determine which of these interventions can be incorporated into routine clinical practice.

The research questions were:

Q1: What level of pain intensity is experienced during peripheral vascular cannulation?

Q2: Which local pharmacological interventions are recommended during peripheral vascular cannulation in adult patients

Q3: What is the clinical effectiveness of these interventions?

Q4: Do these interventions affect other outcomes, such as patient satisfaction, anxiety, or safety, or provide additional benefits?

## 2. Methods

### 2.1. Study Design

This systematic review of the literature was guided by the Joanna Briggs Institute critical appraisal tools for use in JBI Systematic Reviews [17,18], and was conducted following the recommendations of the Preferred Reporting Items for Systematic Reviews and Meta-Analyses (PRISMA) statement [19] (Supplementary Materials).

### 2.2. Search Strategy

The following databases were searched: PubMed, CINAHL, Web of Science, and Scopus. The following keywords were used: adults, outpatients, inpatients, ‘Peripheral Catheterization’, Venipuncture, ‘peripheral intravenous catheter’ OR ‘venous access’ OR ‘PIVC’, ‘IV insertion’, ‘Pain Management’, ‘Analgesia’, ‘Pain Reduction’, ‘Pain Prevention’, ‘Pain relief’, intervention, pharmacological, EMLA, ‘topical anesthetics’. Keywords were entered along with their combinations using AND or OR. Search strategies applied to the search are presented in Table 1. The number of articles found during each search test was limited to studies conducted between 2015 and 2025. The final search was carried out on 25 December 2025.

Two reviewers independently screened all titles and abstracts for eligibility. Full-text articles were independently assessed based on predefined inclusion and exclusion criteria. All publications were analyzed by title and abstract to exclude duplicates and irrelevant entries. Full-text articles were then read and critically evaluated according to the eligibility criteria (PICO framework and inclusion/exclusion criteria). The reference lists of studies identified during the initial search were also screened to ensure that no relevant research was overlooked. The PICO framework and all of the inclusion and exclusion criteria applied to the search are presented in Table 2 and Table 3. Disagreements were resolved through discussion, and when necessary, a third reviewer was consulted.

We included only studies evaluating local pharmacological interventions for pain reduction during peripheral venous cannulation in adults. Studies evaluating exclusively non-pharmacological interventions (e.g., distraction, audiovisual techniques, vibration devices) were excluded.

### 2.3. Data Extraction

Data were extracted by AM, WMD. The following information was extracted: author (year), study design, sample size, setting, intervention, description of intervention, outcome measures, pain scale used, and results.

### 2.4. Data Synthesis

A quantitative meta-analysis was not performed due to substantial clinical and methodological heterogeneity across the included studies. This included the use of different pain assessment scales (NRS, VAS, and VRS), variability in pharmacological agents, differences in application timing and dosage, variation in cannula sizes and clinical settings, and inconsistent outcome reporting. Therefore, pooling of effect sizes and formal statistical heterogeneity assessment (e.g., I^2^ statistics) was not methodologically appropriate, and a structured narrative synthesis was adopted. In addition, a structured narrative approach was selected to enhance clinical interpretability and practical applicability of the findings for routine nursing and clinical practice.

### 2.5. Quality Assessment

Two researchers independently assessed the quality of the included studies. The critical appraisal tools used to assess quality were JBI Checklists for randomized controlled trials [19]. The score was divided into three categories: high, moderate, and low (Table 4). A point was only awarded if the answer to a question from the tool was ‘Yes’. Any discrepancies were resolved through discussion until consensus was reached.

Of the 15 included studies, 10 were classified as high-quality, four as moderate-quality, and one as low-quality according to the JBI critical appraisal tools. The most frequent sources of potential bias were the lack of blinding of participants and personnel, as well as unclear allocation concealment. These methodological limitations may reduce the strength of causal inferences drawn from some studies.

### 2.6. Ethical Approval

Since this study was a systematic review, ethical approval was not required.

## 3. Results

### 3.1. Study Selection

**Figure 1:**
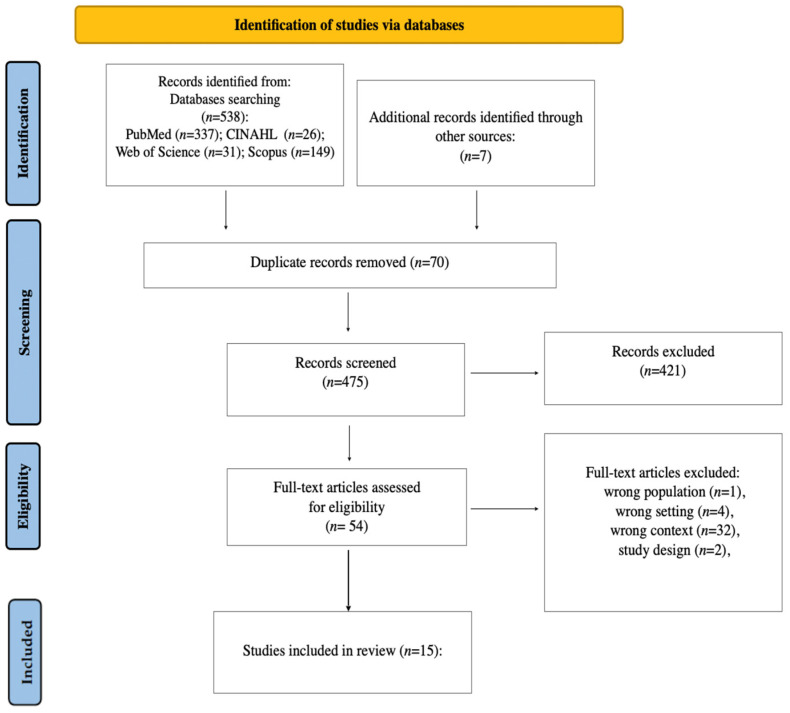
PRISMA 2020 flow diagram of study selection.

The database search resulted in a total of 538 articles. A total of 70 duplicates and 423 articles were removed after abstract review. After full-text analysis of articles, 39 studies were removed due to having the wrong population, wrong setting, wrong context, or study design. A total of 15 studies with randomized controlled trials were included in the analysis (Figure 1). The studies were conducted in Germany (n = 2), USA (n = 5), Iran (n = 3), Japan (n = 2), Denmark (n = 1), Turkey (n = 1) and India (n = 1).

### 3.2. Characteristics of Included Studies

Rather than reproducing the content of Table 5 in narrative form, this section provides a higher-level synthesis of findings across studies, highlighting consistent patterns and clinically relevant trends in pain reduction.

### 3.3. Pain Intensity During Peripheral Venous Cannulation

To improve comparability across studies, results were grouped by pain assessment scale (NRS, VAS, and VRS) and by type of pharmacological intervention rather than directly compared across all studies. This approach reflects differences in scale properties and reporting formats that precluded standardized quantitative pooling. In the studies analyzed above, pain intensity associated with peripheral venous cannulation was most commonly assessed using the Numerical Rating Scale (NRS) and the Visual Analogue Scale (VAS). In control groups receiving placebo or no intervention, pain intensity was generally moderate and ranged from NRS 3 to 6, whereas in groups receiving pain-reducing interventions it ranged from NRS 2 to 4 [2,5,6,13,14]. In groups in which vapocoolant spray, cryospray, or ethyl chloride was used, pain intensity was lower, typically NRS 1–2, compared with NRS 3 to 5 in control groups [2,5,7,9,13]. The use of topical local anesthetics in the form of creams or patches was also associated with low pain scores. In randomized studies, mean pain intensity in intervention groups ranged from NRS 1.5 to 2, compared with approximately NRS 3 in placebo groups [15]. In the study by Yoshida et al., pain assessed using the VAS was 2 in the group receiving a lidocaine–prilocaine patch, whereas a value of 4 was reported in the group receiving injectable lidocaine [6]. Pain intensity was also dependent on the size of the cannula used during the procedure. In a comparative study, pain intensity in the control group was NRS 5 for 17G cannulas and NRS 3 for 20G cannulas. In the intervention group, corresponding values were NRS 2 to 3 and NRS 1 to 2, respectively [2,12].

### 3.4. Pharmacological Interventions Recommended During Peripheral Venous Cannulation

The presented studies evaluated several pharmacological methods aimed at reducing pain during peripheral venous cannulation. The most frequently analyzed interventions included:

1. Cryotherapy, including vapocoolant spray, ethyl chloride and cryospray. These interventions do not require injection and are characterized by a very rapid onset of action [2,3,5,9,13].
2. Topical local anesthetics in the form of creams or patches, including EMLA cream, tetracaine gel, topical ketamine and diclofenac patches. These agents also do not require disruption of skin integrity; however, their effectiveness depends on the duration of application on the skin [2,6,11,16].
3. Injectable lidocaine, which is an effective local anesthetic but requires disruption of skin continuity and may therefore cause additional procedural pain [2,6].

### 3.5. Clinical Effectiveness of the Interventions

1. Interventions involving cryotherapy demonstrated largely consistent and clinically noticeable effects across most studies, with a typical reduction in pain intensity of approximately 1 to 2 points on the NRS. According to published thresholds for minimal clinically important difference (MCID) in acute procedural pain, a reduction of approximately 1.3–2.0 points on numerical or visual analog scales is generally considered clinically meaningful. Therefore, while several interventions achieved changes likely to be perceived as beneficial by patients, some reported differences may represent only marginal clinical improvement.

- Vapocoolant vs. placebo: NRS 2 vs. 4 [13].
- Vapocoolant vs. placebo (venipuncture): NRS 1 vs. 3 [5,7].
- Ethyl chloride vs. placebo: NRS 2 vs. 4 [2].
- Cryospray vs. control group: NRS 1 vs. 3 [9].

It should be noted, however, that not all studies demonstrated a clinically significant effect. In the study by Edwards et al. 2017, the difference between the intervention and placebo groups was small and not clinically significant: NRS 2 vs. 2.5 [10].

2. EMLA cream, lidocaine–prilocaine patch, topical ketamine, injectable lidocaine

- EMLA vs. placebo: 1.66–1.11 vs. 1.9–3.16—effect depending on application time and study population [15].
- Lidocaine–prilocaine vs. injectable lidocaine: VAS 2 vs. 4 [6].
- Topical ketamine: In the study by Heydari et al., both topical ketamine and EMLA produced similar pain scores, NRS 1.7, and were more effective than placebo, NRS 3.16 [3].

### 3.6. Interventions and Their IMPACT on Other Outcomes (e.g., Satisfaction, Anxiety, Safety)/Other Benefits

In the analyzed studies, in addition to pain intensity, other clinical outcomes were also assessed. The most frequently reported outcomes included patient satisfaction, safety of the applied interventions, and their impact on the technical aspects of cannulation [7]. Studies comparing vapocoolant spray, cryospray, or ethyl chloride with placebo or no intervention demonstrated that the use of these methods was associated with higher patient-reported satisfaction and a greater willingness to use the same method again in the future [5,7,9]. In some studies, positive evaluations of the procedure by healthcare staff were also reported, with no significant differences observed in first-attempt success rates or the technical difficulty of cannulation [3,7]. The use of topical local anesthetics in the form of creams or patches, particularly lidocaine–prilocaine (EMLA), was associated with high patient satisfaction and low procedural pain scores; however, some studies indicated a longer preparation time for cannulation due to the need for an adequate application period [16]. In studies comparing lidocaine–prilocaine patches with injectable lidocaine, the transdermal form was rated as more comfortable, mainly due to the absence of additional pain related to anesthetic injection [6]. With regard to safety, the majority of the analyzed studies did not report clinically significant adverse events related to the applied interventions. Vapocoolant spray and cryospray were generally well tolerated, and reported adverse effects were mild and transient [5,9].

## 4. Discussion

The aim of this review was to identify local pharmacological interventions recommended during peripheral venous cannulation to reduce procedural pain, to assess their clinical effectiveness, and to determine which of these interventions may be incorporated into routine clinical practice. The results of the analysis allowed answers to be provided to the formulated research questions. Overall, the observed pattern of modest but generally clinically meaningful pain reduction suggests that the choice of intervention should be guided not only by efficacy but also by feasibility, availability, and workflow integration in routine clinical settings. Common methodological weaknesses across the included studies included small sample sizes, lack of participant and assessor blinding, unclear allocation concealment, and variability in cannula size and clinical context. These limitations increase the risk of bias and reduce confidence in the precision of reported effect estimates.

The analysis of the included studies demonstrated that peripheral venous cannulation in adult patients is most commonly associated with moderate-intensity pain, as confirmed by multiple studies [2,5,10,13]. Pain intensity varied depending on the clinical context and technical parameters, particularly cannula size, with larger cannulas being associated with higher pain levels [2,12]. These findings indicate that cannulation should not be regarded as a pain-neutral procedure.

Various local pharmacological interventions were evaluated in the analyzed studies [2,4]. The lack of a unified standard of care highlights the need to tailor analgesic methods to organizational and clinical conditions. In many countries, nurses have the authority to independently apply selected local-analgesic methods, whereas in Poland the scope of such competencies is more limited, which may hinder the routine implementation of effective interventions.

Most of the analyzed interventions demonstrated significant effectiveness in reducing procedural pain compared with placebo or no intervention. The most consistent effects were observed with the use of vapocoolants and topical local anesthetics in cream or patch form [2,3,5,9,15]. The effectiveness of vapocoolant spray was additionally confirmed in a meta-analysis [2]. Injectable lidocaine also showed an analgesic effect; however, its use was associated with additional pain related to the injection itself [6]. These differences support the need for individualized selection of analgesic interventions.

Beyond pain reduction, the interventions also influenced other clinical outcomes, particularly patient satisfaction and procedure safety [7,9].

The conducted review indicates that local pharmacological interventions are effective and safe in reducing pain during peripheral venous cannulation and may be integrated into routine clinical practice. In particular, vapocoolant sprays and topical anesthetics in cream or patch form appear to be feasible options for widespread use. In the context of the Polish healthcare system, these findings support consideration of expanding nurses’ competencies regarding the independent use of selected local-analgesic methods, which could contribute to improved quality of care, patient comfort, and standardization of procedural pain management. Accordingly, the strength of the recommendations derived from this review should be aligned with the overall quality of the evidence, which ranged from moderate to high but was limited by methodological heterogeneity and risk of bias in several studies.

Our findings are broadly consistent with recent systematic reviews and meta-analyses reporting modest but clinically relevant reductions in pain associated with vapocoolant sprays and topical anesthetics. However, compared with these prior reviews, which often emphasize pooled effect estimates under controlled conditions, the present review highlights greater variability in effectiveness across real-world clinical settings, likely driven by differences in application protocols, staff training, and organizational constraints.

## 5. Limitations

When interpreting the results of this analysis, several limitations should be considered. The included studies differed in terms of study design, types of interventions, duration of application, and pain assessment scales used. The subjective nature of pain scales may have affected the comparability of results. Additionally, studies conducted outside the Polish healthcare system may limit the direct transferability of some interventions to national clinical practice. This systematic review was not registered in PROSPERO, which may increase the risk of reporting bias and reduce methodological transparency. Furthermore, the absence of pooled effect estimates, formal heterogeneity assessment (e.g., I^2^), sensitivity analyses, and evaluation of publication bias limits the precision and generalizability of the conclusions.

## 6. Conclusions

1. Peripheral venous cannulation in adult patients is most commonly associated with moderate-intensity pain and should not be regarded as a pain-neutral procedure.
2. Pharmacological interventions demonstrate significant clinical effectiveness in reducing pain associated with the cannulation procedure.
3. The majority of the analyzed pharmacological interventions are characterized by good patient acceptance and a favorable safety profile.
4. The use of pharmacological interventions prior to peripheral venous cannulation not only reduces pain but also improves patient satisfaction and overall patient experience during hospitalization.

## 7. Implications for Clinical Practice

The results presented in this article indicate that pharmacological methods for reducing pain during peripheral venous cannulation can and should be integrated into the cannulation procedure in routine clinical practice, particularly in high-demand settings such as emergency departments, operating theaters, and surgical wards. Each of the analyzed interventions is characterized by distinct advantages; therefore, consideration of all available options and selection of the most appropriate method for specific patient populations is recommended. In the context of the Polish healthcare system, the findings of this review may serve as a basis for discussion on expanding the competencies of nurses regarding the independent application of selected analgesic methods. Such an approach could significantly contribute to improving the quality of care, increasing patient comfort and satisfaction, and standardizing the management of procedural pain in clinical practice in Poland.

## Figures and Tables

**Table 1:** Search strategy, filters and results.

Databases	Search Strategy
PubMed	(((adults OR outpatients OR inpatients) AND (‘Peripheral Catheterization’ OR ‘Venipuncture’ OR ‘peripheral intravenous catheter’ OR ‘venous access’ OR ‘PIVC’ OR ‘IV insertion’)) AND (‘Pain Management’ OR ‘Analgesia’ OR ‘Pain Reduction’ OR ‘Pain Prevention’ OR ‘Pain relief’)) AND (intervention OR pharmacological OR EMLA OR ‘topical anesthetics’) Limit: Years, adults, language Results: 337
CINAHL	TX (adults OR outpatients OR inpatients) AND TI (‘Peripheral Catheterization’ OR ‘Venipuncture’ OR ‘peripheral intravenous catheter’ OR ‘venous access’ OR ‘PIVC’ OR ‘IV insertion’) AND TX (‘Pain Management’ OR ‘Analgesia’ OR ‘Pain Reduction’ OR ‘Pain Prevention’ OR ‘Pain relief’) AND (intervention OR pharmacological OR EMLA OR ‘topical anesthetics’) Limit: Years, adults Results: 26
Web of Science	(((TS = (adults OR outpatients OR inpatients)) AND TI = (‘Peripheral Catheterization’ OR ‘Venipuncture’ OR ‘peripheral intravenous catheter’ OR ‘venous access’ OR ‘PIVC’ OR ‘IV insertion’)) AND TS = (‘Pain Management’ OR ‘Analgesia’ OR ‘Pain Reduction’ OR ‘Pain Prevention’ OR ‘Pain relief’)) AND TS = (intervention OR pharmacological OR EMLA OR ‘topical anesthetics’) Limit: Years Results: 31
Scopus	(ALL (adults OR outpatients OR inpatients) AND TITLE-ABS-KEY (“Peripheral Catheterization” OR “Venipuncture” OR “peripheral intravenous catheter” OR “venous access” OR “PIVC” OR “IV insertion”) AND TITLE-ABS-KEY (“Pain Management” OR “Analgesia” OR “Pain Reduction” OR “Pain Prevention” OR “Pain relief”) AND ALL (intervention OR pharmacological OR EMLA OR “topical anesthetics”)) AND PUBYEAR > 2014 AND PUBYEAR < 2026 AND (LIMIT-TO (LANGUAGE, “English”)) Limit: Years, language Results: 149

**Table 2:** PICO framework.

	Inclusion Criteria
Population (P)	Adult patients ≥ 18 years old
Intervention (I)	To relieve pain during cannulation
Comparison (C)	Standard/usual care
Outcome (O)	Q1—Type of interventions Q2—Clinical effectiveness: Pain level Q3—Other benefits (e.g., patients’ comfort, satisfaction, anxiety)

**Table 3:** Inclusion and exclusion criteria.

	Inclusion Criteria	Exclusion Criteria
Patients	Adult patients (≥18 years old), Inpatient, outpatient	Pediatric patients (<18 years old),
Intervention	Pharmacological (e.g., EMLA Cream…)	Non-pharmacological interventions
Type of Catheter	PIVC only	Midline, PICC, CVC, HDCVC, Port
Years considered/Time period	All evidence published in the last 10 years, period 2015–2025	Publications prior to 2015
Language	English, Polish	Other languages
Databases	PubMed, CINAHL, Web of Science, Scopus	Other databases Grey literature
Study Type	RCTs, Quasi-experimental Prospective/Retrospective	Quantitative studies Qualitative studies Reviews (any type) Letters to the editor Case reports

**Table 4:** JBI critical appraisal tools—checklist for randomized controlled trials.

Author, Year	1	2	3	4	5	6	7	8	9	10	11	12	13	Total	
Dirk Rush, 2017 [2]	Y	Y	Y	Y	N	N	Y	Y	Y	Y	Y	Y	Y	11/13	H
Farhad Heydari, 2021 [3]	Y	U	Y	Y	Y	Y	Y	Y	Y	Y	Y	Y	Y	12/13	H
Kurt Fossum, 2016 [4]	U	Y	Y	Y	Y	U	Y	Y	Y	Y	Y	Y	Y	11/13	H
Sharon E. Mace, 2015 [5]	Y	Y	Y	Y	Y	Y	Y	Y	Y	Y	Y	Y	Y	13/13	H
Susumu Yoshida, 2025 [6]	N	N	Y	N	N	U	Y	Y	Y	Y	Y	Y	Y	8/13	L
Tracy Barbour, 2017 [7]	Y	Y	Y	Y	Y	Y	Y	Y	Y	Y	Y	Y	Y	13/13	H
Atousa Akhgar, 2025 [8]	Y	Y	Y	N	N	N	Y	Y	Y	Y	Y	Y	Y	10/13	M
Jakob Bjørbaek Pedersen, 2024 [9]	Y	Y	Y	Y	Y	Y	Y	Y	Y	Y	Y	Y	Y	13/13	H
Courtney Edwards, 2017 [10]	Y	Y	Y	Y	Y	Y	Y	Y	Y	Y	Y	Y	Y	13/13	H
Faezeh Babaieasl, 2019 [11]	U	Y	N	Y	Y	Y	Y	Y	Y	Y	Y	Y	Y	11/13	H
Dirk Rush, 2017 [12]	Y	Y	Y	N	N	U	Y	Y	Y	Y	Y	Y	Y	10/13	M
Sharon E. Mace, 2017 [13]	Y	Y	Y	Y	Y	Y	N	Y	Y	Y	Y	Y	Y	12/13	H
Tulay Basak, 2021 [14]	Y	Y	Y	N	Y	N	Y	Y	Y	Y	Y	Y	Y	11/13	H
Tomomi Matsumoto, 2018 [15]	Y	Y	Y	N	N	N	Y	Y	Y	Y	Y	Y	Y	10/13	M
Zempsky William, 2016 [16]	U	Y	Y	Y	U	U	U	Y	Y	Y	Y	Y	Y	9/13	M

Y—Yes; N—No; U—unclear. H = High (if ≥80% of the assessment tool items received a point); M = Moderate (if ≥65% of the assessment tool items received a point); L = Low ≤ 55% (if ≤55% of the assessment tool items received a point). Q1—Was true randomization used for assignment of participants to treatment groups? Q2—Was allocation to treatment groups concealed? Q3—Were treatment groups similar at the baseline? Q4—Were participants blind to treatment assignment? Q5—Were those delivering treatment blind to treatment assignment? Q6—Were outcomes assessors blind to treatment assignment? Q7—Were treatment groups treated identically other than the intervention of interest? Q8—Was follow up complete and if not, were differences between groups in terms of their follow up adequately described and analyzed? Q9—Were participants analyzed in the groups to which they were randomized? Q10—Were outcomes measured in the same way for treatment groups? Q11—Were outcomes measured in a reliable way? Q12—Was appropriate statistical analysis used? Q13—Was the trial design appropriate, and any deviations from the standard RCT design (individual randomization, parallel groups) accounted for in the conduct and analysis of the trial?

**Table 5:** Characteristics of included studies and results.

Author (Year)	Study Design	Sample Sizes	Group Sizes	Setting	Intervention	Outcome Measures	Pain Level	Results
Dirk Rush et al. (2017) [2]	Prospective, randomized, controlled study	N = 160	80 = Lidocaine injection 80 = Vapocoolant spray	University hospital, single-center	Trial of lidocaine infiltration versus vapocoolant spray for reducing discomfort during arterial cannulation.	NRS	Lidocaine NRS= 4.5 Vapocoolant spray NRS= 3.4	+ Vapocoolant spray compared with subcutaneous lidocaine injection provided at least similar effectiveness to mitigate discomfort associated with radial artery puncture.
Farhad Heydari et al. (2021) [3]	Randomized clinical trial	N = 300	100 = Topical ketamine cream 100 = EMLA cream 100 = Placebo cold cream	Emergency department of Alzahra and Kashani Hospital, two university teaching hospitals in Isfahan	Trial of 10% ketamine cream versus 5% EMLA cream versus placebo for venipuncture analgesia.	NRS	Ketamine NRS = 1.72 EMLA NRS = 1.66 Placebo cold cream NRS = 3.16	+ Local cutaneous ketamine is as effective as EMLA in relieving pain during venipuncture.
Kurt Fossum et al. (2016) [4]	Randomized, double-blind, placebo-controlled, crossover trial	N = 38	8 + 13 = Topical ethyl chloride (left arm and right arm) 11 + 6 = Sterile water aerosol spray (left arm and right arm)	Urban tertiary-care hospital	A randomized trial enrolled emergency department providers to compare ethyl chloride with sterile water spray, with catheterization in either antecubital fossa and a 5 min washout between procedures.	NRS	Topical ethyl chloride NRS = 2 Sterile water spray NRS = 4	+ Topical ethyl chloride yields a greater reduction in pain associated with venous catheterization compared with topical placebo.
Sharon E. Mace (2015) [5]	Prospective, double-blind, randomized, controlled trial	N = 100	50 = Vapocoolant spray 50 = Placebo spray	Hospital emergency department or observation unit	Trial of vapocoolant spray and placebo spray.	NRS	Vapocoolant spray NRS = 1 Placebo spray NRS = 3	+ Compared with a placebo spray, there was a significant decrease in the pain of venipuncture on an NRS.
Susumu Yoshida et al. (2025) [6]	Prospective observational study	N = 70	35 = Lidocaine–prilocaine patch 35 = Lidocaine injection	Operating room	Trial of 2% lidocaine injection versus lidocaine–prilocaine patch for reducing pain during peripheral venous catheter insertion.	VAS	Lidocaine–prilocaine patch VAS = 2 Lidocaine injection VAS = 4	+/− No difference was observed in pain intensity during PVC insertion between lidocaine–prilocaine patch and 2% intradermal lidocaine; however, VAS scores for anesthetic application were lower with the patch, providing equivalent analgesia without injection pain.
Tracy Barbour et al. (2017) [7]	Prospective double-blind, randomized controlled trial	N = 100	50 = Vapocoolant spray 50 = Placebo spray	Emergency department observation unit	Trial of vapocoolant spray versus sterile-water placebo spray for reducing pain during blood draws performed with a standardized vacutainer technique.	NRS	Vapocoolant spray NRS = 1 Placebo spray NRS = 3	+ Less pain when a vapocoolant spray was used in adults undergoing venipuncture in the emergency department.
Atousa Akhgar et al. (2025) [8]	Randomized clinical trial study	N = 80	40 = Lidocaine–prilocaine cream 40 = Vapocoolant spray	Emergency department of an academic hospital in Iran	Trial of vapocoolant spray versus lidocaine–prilocaine cream for pain reduction during intravenous cannulation using a 20-gauge catheter.	NRS	Lidocaine–prilocaine cream NRS = 3 Vapocoolant spray NRS = 2	+/− The vapocoolant spray was not statistically more effective than lidocaine–prilocaine cream in pain reduction during intravenous cannulation.
Jakob Bjørbaek Pedersen et al. (2024) [9]	Prospective randomized placebo-controlled trial	N = 130	64 = Cryospray 64 = Control group	General operating theater at Hospital Sønderjylland, Southern Denmark	Trial of cryospray versus placebo for reducing patient-reported pain during venous cannulation.	NRS	Cryospray NRS = 1 Control group NRS = 3	+ Cryospray significantly reduced pain during venous cannulation without increasing procedure difficulty. Patients reported lower pain scores and a greater preference for cryospray in future procedures.
Courtney Edwards et al. (2017) [10]	Randomized, double-blind, placebo-controlled, single-center trial	N = 71	38 = Topical vapocoolant spray 34 = Control group	Adult emergency department	Trial of vapocoolant spray versus placebo for pain and anxiety relief during peripheral intravenous cannulation.	NRS	Topical vapocoolant spray NRS = 2 Control group = 2.5	− Among adult patients in the emergency department, no significant differences in pain relief or alleviation of anxiety were found between treatments using a vapocoolant spray or placebo during PIV cannulation.
Faezeh Babaieasl et al. (2019) [11]	Double-blind, randomized controlled trial	N = 154	61 = EMLA patch 50 = Diclofenac patch 46 = Control group	Cardiology and coronary care unit of an educational hospital in Babol, Northern Iran	Trial of EMLA patch versus diclofenac (TDP) patch versus placebo for reducing pain and phlebitis from peripheral intravenous catheterization.	VAS	EMLA VAS = 38.77 ± 23.28 Diclofenac VAS = 39.40 ± 21.60 Control group VAS = 86.41 ± 22.49	+ EMLA and TDP had similar effects on reducing the pain of IV cannulation, but the phlebitis rate was lower following the use of TDP.
Dirk Rush et al. (2017) [12]	Randomized, controlled trial	N = 450	75 = Injected lidocaine (17G) 75 = Injected lidocaine (20G) 75 = Vapocoolant spray (17G) 75 = Vapocoolant spray (20G) 75 = Control group (17G) 75 = Control group (20G)	Marburg University Medical Center	Trial of intradermal 2% lidocaine versus vapocoolant spray versus placebo for pain reduction during venipuncture with 17G or 20G cannulas.	NRS	Injected lidocaine (17G) NRS = 3.2 Injected lidocaine (20G) NRS = 3.5 Vapocoolant spray (17G) NRS = 2.6 Vapocoolant spray (20G) NRS = 2.1 Control group (17G) NRS = 5.0 Control group (20G) NRS = 3.0	+ The present results underline the indication for local anesthetic pretreatment if a venous cannula of 17G or larger is inserted on the dorsum of the hand. Cryoanesthesia may offer advantages in this setting, compared with the thus far more common lidocaine infiltration, in terms of condition of the puncture site, effectiveness, and simplified processes. In smaller venous cannulas (20G and smaller), positive effects are statistically significant.
Sharon E. Mace (2017) [13]	Prospective, randomized, double-blind controlled trial	N = 300	150 = Vapocoolant spray 150 = Sterile water placebo spray	Urban, academic, tertiary-care referral hospital	Trial of vapocoolant spray versus sterile-water placebo for reducing pain during peripheral intravenous cannulation.	NRS	Vapocoolant spray NRS = 2 Sterile water placebo spray NRS = 4	+ The median NRS interquartile range for PIV cannulation pain was 4 (2–7) for the placebo spray group vs. 2 (0–4) for the vapocoolant spray group.
Tulay Basak et al. (2021) [14]	Single-blinded, randomized controlled study	N = 88	44 = Vapocoolant spray 44 = Control group	Regional Blood Center of a state hospital in Gulhane Military Medical Academy.	Trial of vapocoolant spray versus no intervention for reducing venipuncture pain in healthy male blood donors.	VAS	Vapocoolant spray VAS = 1.90 Control group VAS = 3.23	+ The study result showed that the use of vapocoolant spray for pain management is an effective method of reducing pain related to venipuncture during the process of blood donation in young male donors.
Tomomi Matsumoto et al. (2018) [15]	Single-center, prospective, randomized, interventional study	N = 24	12 = EMLA cream 12 = Lidocaine tape	Tomakomai City Hospital, Hokkaido, Japan	Trial of EMLA cream versus lidocaine tape for reducing venipuncture pain prior to induction of general anesthesia.	VAS, VRS	EMLA cream VAS, VRS = 4/2 Lidocaine tape VAS, VRS = 17/2	+ The study results indicate that EMLA cream is more effective for pain relief during venipuncture than lidocaine tape.
Zempsky William et al. (2016) [16]	Randomized, double-blind, placebo-controlled	N = 693	345 = Powder lidocaine 348 = Control group	NO DATA	Trial of needle-free powder lidocaine versus sham placebo for pain reduction during venipuncture or venous cannulation in adults.	VAS	Power lidocaine VAS = 74.2 Control group VAS = 62.1	+ Use of a needle-free powder lidocaine delivery system resulted in a significant reduction in pain during venipuncture and peripheral intravenous cannulation in adults.

Legend: NRS—Numerical Rating Scale; VAS—Visual Analogue Scale; VRS—Verbal Rating Scale; EMLA—Eutectic mixture of local anesthetics; TDP—Transdermal patch; [+]—Positive effect; [−]—No effect.

## Data Availability

The authors declare that the data of this research are available from the correspondence author on request.

## Supplementary Materials

The following supporting information can be downloaded at: https://www.mdpi.com/article/10.3390/jcm15031262/s1, Table S1: PRISMA 2020 Checklist.
